# Re-evaluation of the yield response to phosphorus fertilization based on meta-analyses of long-term field experiments

**DOI:** 10.1007/s13280-017-0971-1

**Published:** 2017-11-20

**Authors:** Uwe Buczko, Michael van Laak, Bettina Eichler-Löbermann, Wolfgang Gans, Ines Merbach, Kerstin Panten, Edgar Peiter, Thomas Reitz, Heide Spiegel, Sabine von Tucher

**Affiliations:** 1 Department of Landscape Ecology and Site Evaluation, University of Rostock, 18059 Rostock, Germany; 2 Department of Crop Science, University of Rostock, 18059 Rostock, Germany; 3 Martin Luther University Halle-Wittenberg, Betty Heimann Str. 3, 06120 Halle (Saale), Germany; 4 Helmholtz-Centre of Environmental Research – UFZ, Hallesche Straße 44, 06246 Bad Lauchstädt, Germany; 5 Institute for Crop and Soil Science, Julius Kühn Institute, Federal Research Centre for Cultivated Plants, Bundesallee 50, 38116 Brunswick, Germany; 6 Plant Nutrition Laboratory, Institute of Agricultural and Nutritional Sciences, Martin Luther University Halle-Wittenberg, 06099 Halle (Saale), Germany; 7 Helmholtz-Centre for Environmental Research – UFZ, Theodor-Lieser-Str. 4, 06120 Halle (Saale), Germany; 8 Austrian Agency for Health and Food Safety – AGES, Spargelfeldstraße 191, 1220 Vienna, Austria; 9 Emil-Ramann-Straße 2, 85354 Freising, Germany

**Keywords:** CART, Crop yield, Fertilization, Phosphorus, Plant-available soil phosphorus

## Abstract

Phosphorus (P) fertilizer recommendations in most European countries are based on plant-available soil P contents and long-term field experiments. Site-specific conditions are often neglected, resulting in excessive P fertilizer applications. P fertilization experiments including relevant site and soil parameters were evaluated in order to analyze the yield response. The database comprises about 2000 datasets from 30 field experiments from Germany and Austria. Statistical evaluations using a classification and regression tree approach, and multiple linear regression analysis indicate that besides plant-available soil P content, soil texture and soil organic matter content have a large influence on the effectiveness of P fertilization. This study methodology can be a basis for modification and specification of existing P fertilization recommendations and thus contribute to mitigate environmental impacts of P fertilization.

**Electronic supplementary material:**

The online version of this article (10.1007/s13280-017-0971-1) contains supplementary material, which is available to authorized users.

## Introduction

Phosphorus (P) is one of the major macronutrients for plant growth and adequate P fertilization is essential to attain optimum yields (Smil 2000). On the other hand, excessive P fertilization is undesirable because P is a scarce, non-renewable resource (Cordell et al. 2009; Vaccari 2009; Schoumans et al. 2015; Mew 2016) and diffuse P losses from excessively fertilized fields are a major cause of eutrophication in surface waters (Correll 1998; Buczko and Kuchenbuch 2007).

In most countries, P fertilizer recommendations are based on the expected nutrient uptake by crops (expected yield × expected P concentration of crop) and the plant-available P content in the soil (Jordan-Meille et al. 2012). The procedure of deducing phosphorus fertilizer recommendations entails three steps (Jordan-Meille et al. 2012): (i) Extraction of plant-available soil P, (ii) calibration of those soil test results, (iii) deducing recommended P fertilizer amounts.

There is no standard definition of “plant-available” P in the soil. Therefore, the term “plant-available” is not defined unambiguously, and for the estimation of plant-available P in the soil, a large variety of extraction methods are in use. In Europe and worldwide, the recommended extraction procedures for the determination of plant-available P differ among countries (Neyroud and Lischer 2003; Jordan-Meille et al. 2012). In Germany, 14 of the 16 federal states base their fertilizer recommendations on the calcium acetate lactate (CAL) extraction method, which employs a solution of calcium lactate, calcium acetate, and acetic acid (Schüller 1969). In two federal states the double lactate (DL) method is used (Riehm 1942). In the calibration step, plant-available P contents are categorized into several classes (in Germany and many other countries five classes), which are interpreted in terms of nutrient supply. These calibrations are mostly based on long-term fertilization trials (Kuchenbuch and Buczko 2011). However, the database used for the calibration step is mostly not accessible in the international literature, and even in countries which use the same extraction procedure, the boundaries of the nutrient availability classes may diverge considerably (Jordan-Meille et al. 2012). This holds true even for various federal states of Germany (Römer 2015).

Various studies have shown that P fertilization recommendations in Germany and several other European countries have been too high in the past several years (Ott and Rechberger 2012; Tóth et al. 2014; Withers et al. 2014), and consequently, the boundaries of the P fertility classes have been too high (VDLUFA 2015). Moreover, besides plant-available soil P contents, other factors have an influence on yield (Kuchenbuch and Buczko 2011), for instance pH value, soil organic carbon content, clay content, weather, and climate parameters. In Germany, the boundaries of the fertility classes (“A” with the lowest, and “E” with the highest contents, whereas the intermediate class “C” is considered optimal for crop growth) are based on yield and soil test data from long-term field experiments, but in general, the calibration procedures are not published. Often, the boundaries are set according to practical considerations and changed with time (Übelhör and Hartwig 2012).

Although in Germany and other countries there is a large number of long-term field experiments dealing with the effects of P fertilization on crop yields (e.g., Baier et al. 2001; Spiegel et al. 2001; Merbach and Schulz 2012), the results of those field fertilization trials have mostly not been compiled or evaluated and analyzed as a whole in the form of a meta-study. In a previous meta-analysis, Kuchenbuch and Buczko (2011) evaluated mainly the results of fertilization trials gained from published, openly accessible sources, whereas large databases remain unpublished at various institutions.

Since in large databases, information about the relationships among the data is often not available prior to analysis, and classification and regression tree approaches (CART, Sonquist and Morgan 1964; Sutton 2005; Strobl et al. 2009) are commonly used in such analyses. CART approaches are non-parametric and do not require any previous assumptions about the distributions or linearity of the variables. Furthermore, they are resistant to outliers and both categorical and numerical predictor variables can be combined. They are relatively easy to use and interpretation of the resulting trees is straightforward. These methods are applicable in many cases when classical parametric methods are not applicable, for instance in cases with many predictor variables but relatively few datasets (Sonquist and Morgan 1964; Strobl et al. 2009). In agricultural sciences, CART approaches have been used to predict how yield is influenced by soil and management factors (Lapen et al. 2001; Lobell et al. 2005; Zheng et al. 2009; Kuchenbuch and Buczko 2011).

The objectives of this work were to compile a large database of long-term P fertilization experiments from Germany and Austria with special emphasis on data which have until now not been published. A meta-analysis of this data was conducted including statistical methods to evaluate the influence of various site-specific soil and environmental factors on the effectiveness of P fertilization.

## Materials and methods

### Compilation of database

**Fig. 1:**
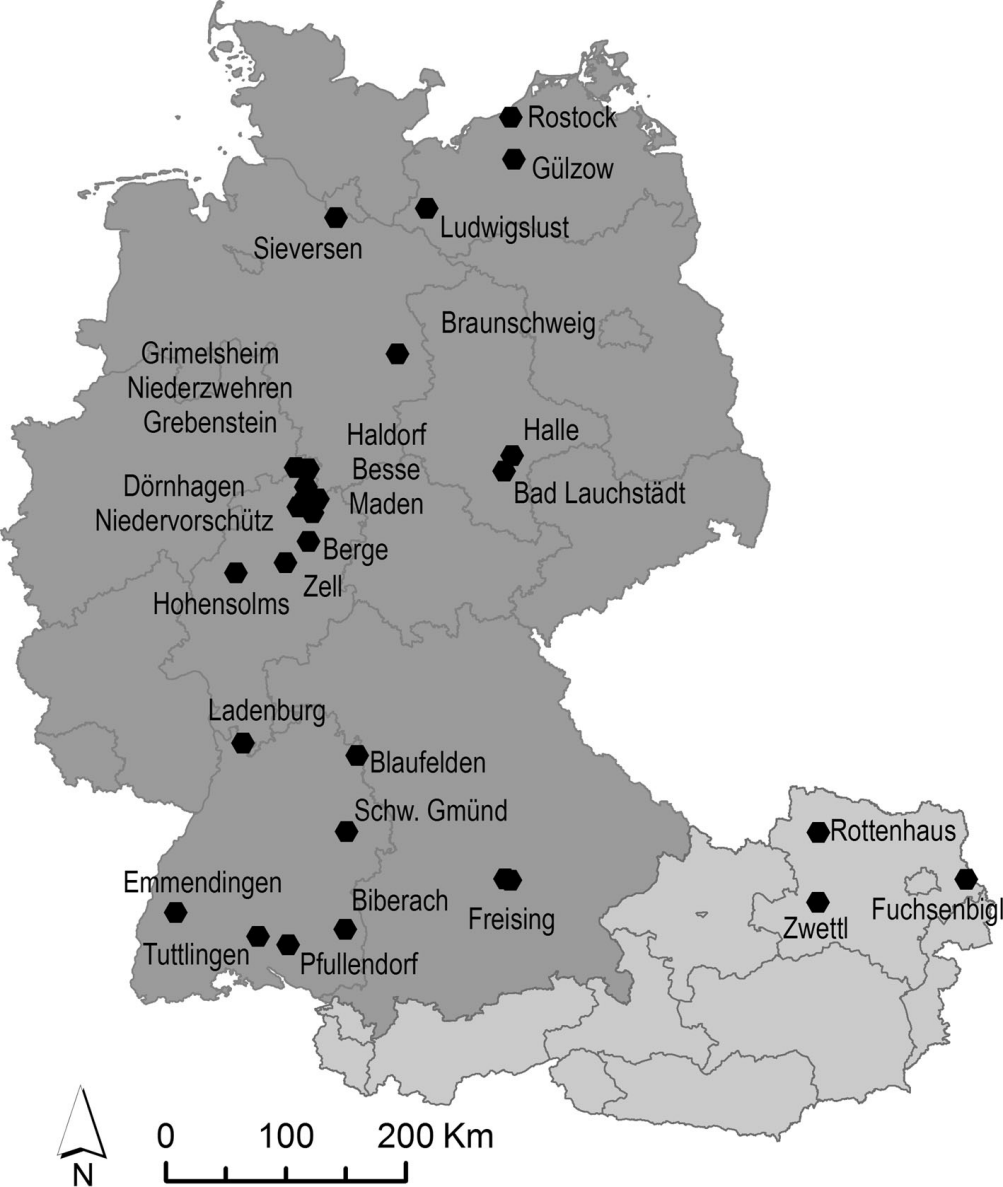
Location of the fertilization trials in Germany and Austria used in this study

Data of phosphorus fertilization trials across Germany and Austria were compiled (Fig. 1) (Table 1). All field experiments focused on the effect of P fertilization on yields and are therefore one-factorial fertilization trials with application rates ranging between 10 and 210 kg P ha^−1^ yea^−1^, i.e., 30–2000% of P export by crops (calculated from actual crop yields and literature data for P contents of various crop types) (Table 2). Effects of fertilizer application rates on yields were compared by calculating the relative yield increases (YI in %) from the ratio of the yield of the fertilized treatment (*y*_f_) and that of the zero fertilization (control) treatment (*y*_0_):

$$ {\text{YI}} = \left( {\left( {y_{\text{f}} /y_{0} } \right){-}1} \right) \times 100 $$

i.e., YI is the percentage value of the increase in crop yield of the fertilized treatment compared with the corresponding control treatment.

**Table 1:** Compilation of fertilization trials used in this study

Site (state)	Duration^a^	*N*	P(CAL,DL)^b^ (mg P 100 g^−1^ soil), in year of trial establishment	pH	*C*_org_ (%)	Clay (%)	Soil type
Berge (He)	1973–1996	69	5.4	6.7	1.6	22	Luvisol
Besse (He)	1986–1996	30	9.9	6.4	1.3	25	Luvisol
Biberach (BW)	1986–1993	24	11	5.6	–	9	Luvisol
Blaufelden (BW)	1984–1993	24	4.0	6.3	–	21	Luvisol
Braunschweig (LS)	1986–1996	109	3.4	5.3	1.3	13	Cambisol
Dörnhagen (He)	1984–2008	74	4.7	6.5	1.0	15	Luvisol
Emmendingen (BW)	1984–1993	30	8.4	7	–	24	Luvisol
Freising (Dürnast) 16 (Ba)	1978–2014	74	4.5	6.1	1.1	24	Luvisol
Freising (Dürnast) 021 (Ba)	1980–2014	105	6.6	6.2	1.1	20	Luvisol
Freising (Dürnast) 022 (Ba)	1980–2014	102	6.9	6.1	1.3	20	Luvisol
Fuchsenbigl (NÖ)	1976–2003	199	17.0	7.5	1.1	18	Chernozem
Grebenstein (He)	1973–2008	99	9.3	6.4	1.1	15	Luvisol
Grimelsheim (He)	1997–2003	21	6.4	6.8	1.8	24	Luvisol
Gülzow (MV)	1998–2014	96	8.8	6	0.7	7.3	Luvisol
Haldorf (He)	1973–2008	81	8.0	6.7	1.4	10.9	Luvisol
Halle (SA)	1979–2002	114	6.7	5.8	1.5	13	Luvisol
Hohensolms (He)	1996–2005	70	5.3	5.3	1.6	21	Cambisol
Ladenburg (BW)	1984–1993	27	6.2	6.2	–	21	Luvisol
Lauchstädt (SA)	1991–2010	20	3.3	5.8	2.1	21	Chernozem
Ludwigslust (Lehsen) (MV)	1995–2004	57	5.1	6.3	0.9	9	Luvisol
Maden (He)	1981–1988	32	9.0	6.1	1.3	18	Luvisol
Niedervorschütz (He)	1974–1985	48	12	7	1.1	16	Luvisol
Niederzwehren (He)	2007–2008	6	5.6	6.7	1.1	13	no data
Pfullendorf (BW)	1984–1993	30	9.7	6.1	–	21	Luvisol
Rostock (MV)	1999–2014	28	3.5	5.6	1.0	3	Cambisol
Rottenhaus (NÖ)	1976–2003	190	6.2	6.5	1.4	30	Cambisol
Schwäbisch Gmünd (BW)	1984–1993	27	11	6.2	–	21	Luvisol
Sieversen (LS)	1998–2003	36	9.0	6.2	1.9	5.5	Cambisol
Tuttlingen (BW)	1984–1992	24	3.1	6.3	–	35	Luvisol
Zell (He)	2006–2008	9	8.2	6	1.8	17	no data
Zwettl (NÖ)	1976–2003	163	7.9	5.4	1	16	Cambisol

*Ba* Bavaria, *BW* Baden-Württemberg, *He* Hesse, *LS* Lower Saxony, *MV* Mecklenburg-Western Pomerania, *NÖ* Lower Austria, *SA* Saxony-Anhalt, *N* number of data

^a^“Duration” here refers to the data utilized for the meta-analysis and does not necessarily coincide with the total duration of the field experiment; for information about the total duration of the field experiments, please refer to Table A1 in the appendix

^b^Average values of the first trial year that was used in our analysis; for the data from MV and SA, the DL extraction procedure was used; for all other the CAL extraction, it was assumed that P(CAL) = P(DL) (Neyroud and Lischer 2003); samples were extracted usually from 0 to 30 cm soil depth

**Table 2:** Ranges of most important soil and fertilization parameters across all field sites

Parameter	Min.	Median	Mean	Max.
Clay content (%)	5.5	18.0	18.0	35.0
Soil organic matter (SOM) (%)	1.2	2.0	2.1	3.6
pH	4.4	6.3	6.3	7.8
Plant-available P in soil (P-DL or P-CAL) (mg P 100 g^−1^)^a^	1.3	8.3	10.0	53.2
P fertilization (kg P ha^−1^ year^−1^)	9.8	43.0	60.7	209.8
Rel. P fertilizer addition [P addition/P export (%)]	29.5	157.8	261.3	2017

^a^Soil P content of all fertilized plots for all years

Soil test values and site-specific factors (pH, organic carbon content, and clay content) are summarized in Tables 1 and 2. Note that this parameter list does not encompass all parameters which probably have an influence on the effectiveness of P fertilization on yields. However, in most of the trials, such parameters were not measured or recorded (for instance, sorption capacity, content of Fe- and Al-oxides, P content in subsoil, root density, cation exchange capacity).

Tables 1 and 2 show that the studied soils have rather high soil P contents. Nevertheless, P fertilizer application rates are very high: in 50% of the data, more than 158% of the P was exported by harvested crops (Table 2).

The fertilization trials have mostly been conducted over many years (see Table S1), and the duration of the experiments utilized in the present meta-analysis is in some cases longer than 20 years (Table 1). The most frequent soil types were Luvisols. The crop rotations are dominated by the crops grown most commonly in Germany, i.e., winter wheat (*n* = 568), winter barley (*n* = 305), summer barley (*n* = 202), sugar beet (*n* = 200), potato (*n* = 197), and oilseed rape (canola) (*n* = 129) (see also Fig. 5).

### Data analysis

The aim of this study was to evaluate the effectiveness of P fertilization on crop yields for a given soil P content. In a similar manner to a previous meta-analysis (Kuchenbuch and Buczko 2011), the data of the field trials were analyzed with a classification and regression tree (CART) approach. This methodology is based on splitting the dataset into segments with a distinct factor combination. As in other CART approaches (Strobl et al. 2009), the impact of several predictor variables on a dependent variable is analyzed by successive binary splits. To determine which predictor variable is best to be used for the split and to calculate the corresponding value of the split point for every allowable split on each predictor variable, the within-segment and between-segment sums of squares are calculated. The split (i.e., predictor variable and split point) which yields the most homogeneous binary split in terms of the dependent variable (i.e., with the largest between-segment and smallest within-segment sum of squares) is chosen for the splitting. The result of the analysis is a binary tree diagram. The endpoints of this tree are relatively homogeneous subgroups of the data. The resulting trees are easy to interpret, since the successive binary splits indicate the relative importance of the predictor variables in explaining the dependent variable. However, as with most other statistical methods, the results provide no information about the processes governing the effect of the influencing variables on the dependent variable. Consequently, the results of this procedure should be complemented by expert knowledge, hypotheses, and further statistical methods. Therefore, multiple linear regression analyses were conducted for comparison (Lobell et al. 2005). Both the CART and regression analyses were done using the program SPSS (version 20.0).

For both the CART and regression analyses, the dependent variable was the relative yield increase (YI), and the influencing factors (predictor variables) were plant-available soil P content (soil test phosphorus, STP), clay content, organic carbon content, pH value, relative P fertilizer application rate, and crop species.

A concern when analyzing time-series data with regression models and regression trees is serial correlation, i.e., the data are auto-correlated in time and therefore not independent. Although the regression coefficients remain unaffected by serial correlation, standard errors may be underestimated (and significances overestimated) when serial correlation occurs (Durbin and Watson 1950; Verbeek 2004). This will lead to the conclusion that the parameter estimates are more precise than they actually are. Since the data used in this study are partly in the form of time-series, we tested for serial correlation using the Durbin–Watson test (Durbin and Watson 1950; Verbeek 2004). This yielded partially Durbin–Watson values < 1, which indicates serial correlation. Consequently, one must be aware that significances of regression tree analysis and linear regression may be slightly overestimated due to serial correlation.

## Results and discussion

**Fig. 2:**
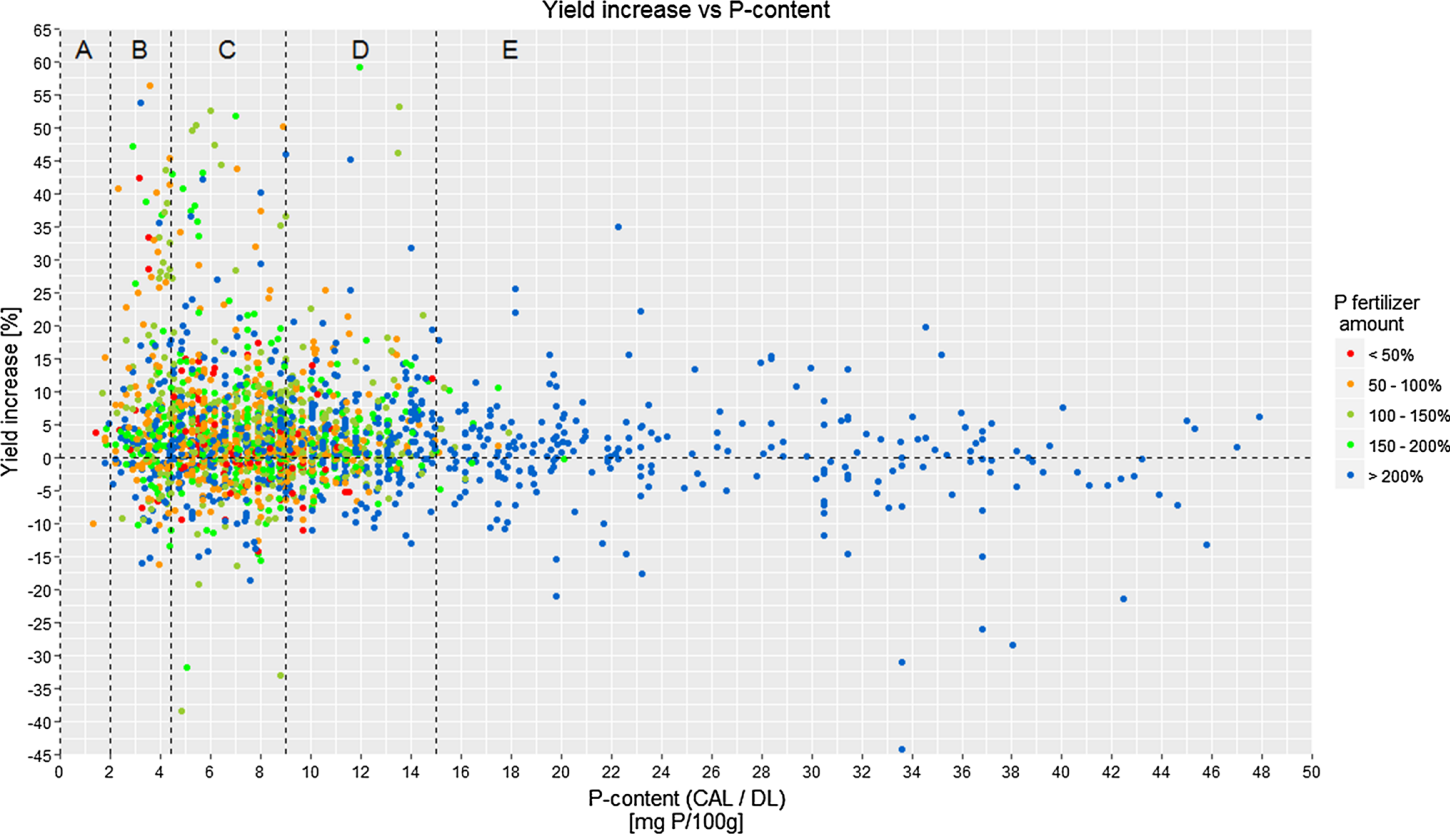
Rel. yield increase (YI) versus soil P content (CAL or DL); fertilizer application rate (“fertilizer amount”) expressed as % of P export by harvested crop; P content classes according to VDLUFA (1997)

The relation between STP and YI for all data points (Fig. 2) reveals highest YI for the soil P content class B and lower YI for higher P content classes (for the boundaries of the classes, see Fig. 2). Similarly, the variability of YI is highest for P content class B and decreases towards class E. When YI values are averaged for each of the five P content classes (Table 3), there are statistically significant differences between the P content classes. Whereas the highest average YI by P fertilization was observed for the P content class B, for P content class A, statistically valid numbers cannot be calculated due to the exceedingly low number of data (*n* = 10). Due to the large variability of YI values observed for all soil P classes, it is difficult to confirm or reject the actual boundaries of soil P classes based on these data alone.

**Table 3:** Rel. yield increase (YI) by P fertilization versus soil P content class (VDLUFA 1997), pH class, SOM content, and fertilizer type; *N* number of data

P content class	A	B	C	D	E
Mean YI	3.46	6.53	4.30	3.61	0.70
*N*	10	268	903	573	256
Significance^a^	(abc)	a	b	b	c
pH class^b^	A	B	C	D	E
Mean YI	7.40	4.71	2.52	2.66	1.29
*N*	275	849	423	167	296
Significance^a^	a	b	c	bc	c
SOM class (%)^c^	≤ 1.5	> 1.5–2	> 2–2.5	> 2.5–3	> 3
Mean YI	2.26	2.60	5.24	8.10	3.86
*N*	96	847	527	234	119
Significance^a^	ab	a	b	c	ab
Fertilizer type	Superphosphate	Triple superphosphate	Hyperphosphate	Thomas phosphate	Others
Mean YI	5.10	2.36	1.31	5.06	4.28
*N*	839	632	119	304	68
Significance^a^	a	b	b	a	ab

^a^Small letters denote statistical significance of differences between groups (Tukey HSD post hoc test, *p* < 0.05)

^b^According to VDLUFA (2000); the corresponding actual pH values depend on soil texture and SOM. For SOM < 4%, the optimum class C corresponds for sandy soil texture to pH values 5.4–5.8. This range gradually increases with clay content up to 6.4–7.2 for a clayey loam to clay soil

^c^Arbitrary SOM classes, since existing SOM classes (e.g., VDLUFA 2000) do not distinguish for SOM < 4%

For all soil P classes, the large number of data points with negative YI, i.e., yield depressions, is striking. This is a phenomenon commonly observed in long-term fertilization trials (e.g., Köster and Schachtschabel 1983; Jungk et al. 1993; Römer 2009; Kuchenbuch and Buczko 2011). Since the yield depressions observed here are more or less equal for all soil P content classes (A: 20%, B: 30.6%, C: 29%, D; 30.5%, E: 41% of datasets), and soil P toxicity is rare under field conditions (Zorn et al. 2013; Lambers and Plaxton 2015), the negative YI cannot reasonably be explained directly by the effect of P fertilization. However, indirectly, high levels of plant-available P (as provided by mineral fertilizer) in general reduce root density (Forde and Lorenzo 2001), and the development of mycorrhiza (Mäder et al. 2000; Williams et al. 2017). This could have a negative impact on the uptake of water and other nutrient elements, for instance the micronutrients Zn and Cu, thus reducing the yield of fertilized treatments.

**Fig. 3:**
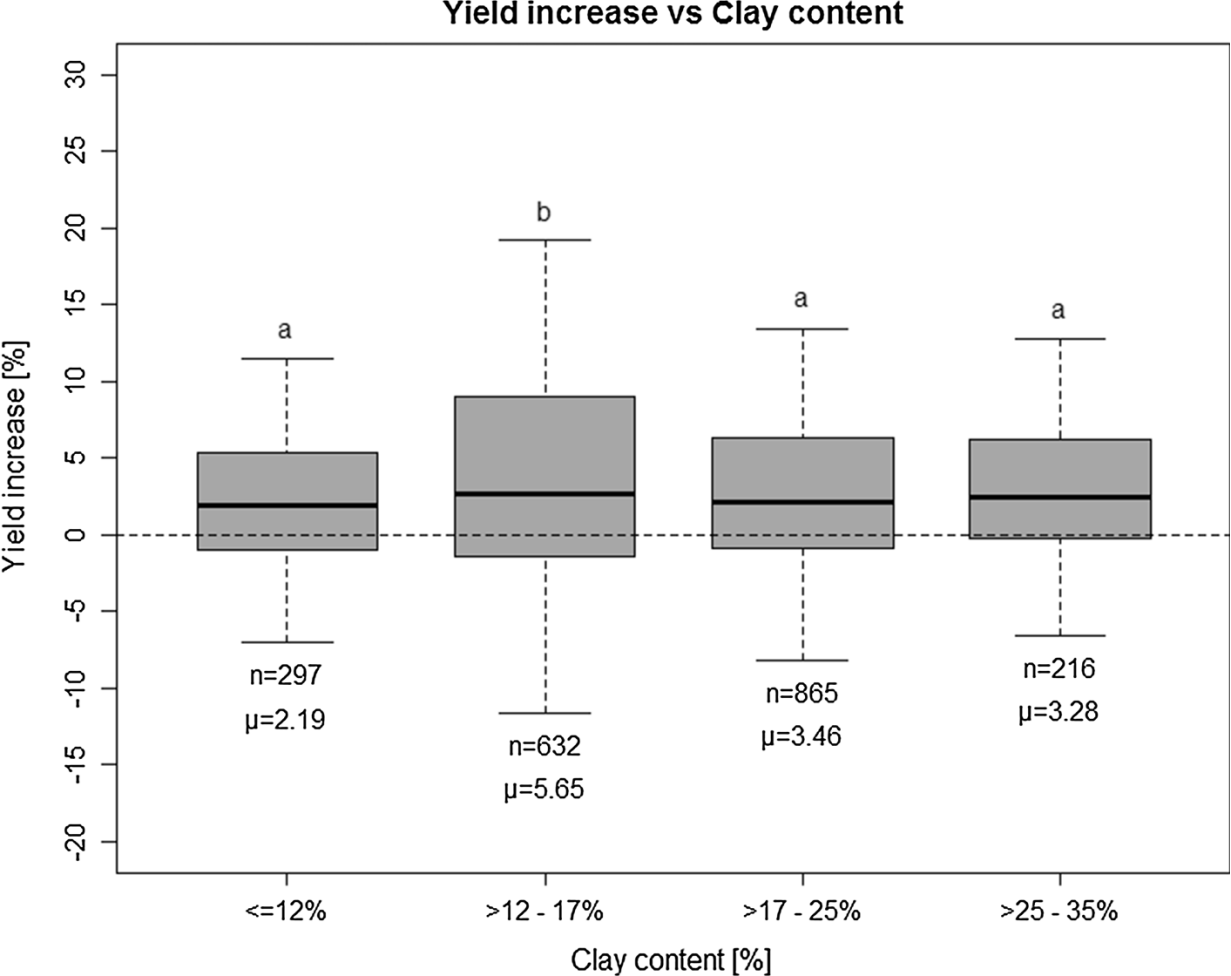
Rel. yield increase (YI) vs clay content class (VDLUFA 1997); boxes delineate the interval between 25- and 75-percentile; horizontal bold lines in the center of the boxes denote median values. Arithmetic averages (*µ*) and number of data (*n*) are given below the boxes. Whiskers delineate the 1.5 fold of the interquartile range (IQR). Outliers (> 1.5 × IQR) are not shown. Small letters above the whiskers denote statistical significance of differences between groups (Tukey HSD post hoc test, *p* < 0.05)

Relative yield increases by P fertilization as a function of soil pH class (Table 3) show highest YI values for pH classes A and B (i.e., acid conditions with low pH values, < 6), and lowest values for class E (high pH values, > 7). This is probably connected with the direct correlation between STP and pH values (Pearson *r* = 0.41). At low pH values (< 5.5), P is strongly adsorbed (e.g., by Fe- and Al-Oxides) in soils and therefore less readily plant available (e.g., von Tucher et al. 2016). Additionally, soil pH influences the availability of other essential plant nutrients and soil microorganisms and might therefore cause yield effects not investigated in the evaluated phosphorus experiments. There is no clear relation between clay content and YI (Fig. 3). On average, YI is highest for the clay content class of 12–17%.

In line with these findings, the interpretation of the effect of soil clay content on plant availability of soil P is not straightforward: in general, the mobility of nutrient ions (especially in the vicinity of roots) is lower in clay-rich soils because the effective diffusion coefficient decreases with increasing clay content, mainly due to sorption on clay surfaces (Jungk and Claassen 1986; Hinsinger et al. 2009). Consequently, the mobility of nutrient ions is reduced in clay-rich soils. In contrast to most nutrients which occur as cations, the negatively charged phosphate anion is predominantly adsorbed to surfaces of Fe-, Mn-, and Al-oxides. These usually constitute only a minor part of the clay fraction, compared with clay minerals (Jungk and Claassen 1986).

YI increases with SOM content, and highest YI values are observed for SOM contents of 2.5–3% (Table 3). However, for SOM > 3%, YI is again significantly lower (but the number of data in that group is lower than in the other groups). In general, P availability is directly correlated with SOM contents, because adsorption of P is reduced by organic anions such as citrate or malate which compete with phosphate anions for adsorption sites at Fe and Al oxide surfaces (e.g., Hunt et al. 2007; Gerke 2015). This may explain the higher effect of P fertilization with higher SOM contents observed for our data. Moreover, SOM contents are correlated with clay contents (not shown here in detail). However, the lower YI for the highest SOM class is not entirely clear. One possible explanation is, that at high SOM contents the release of available P from organic P compounds is more important than for lower SOM contents.

**Fig. 4:**
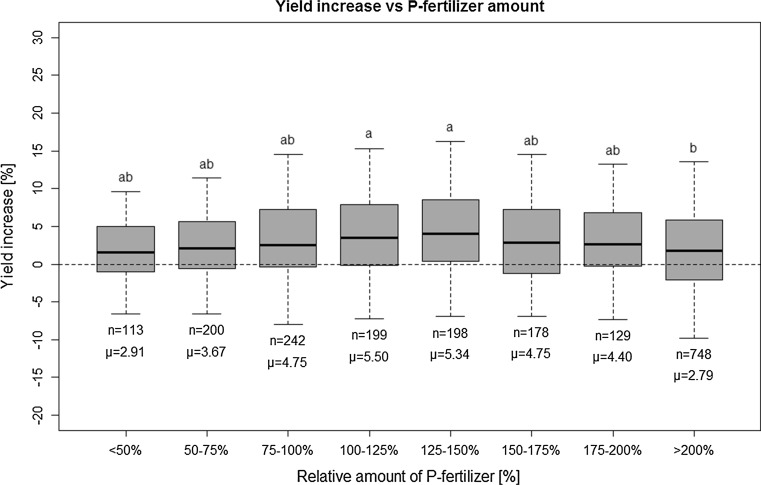
Rel. yield increase (YI) versus rel. P fertilizer amount (i.e., P fertilizer addition/P export by harvested crop × 100); for the meaning of symbols, please refer to Fig. 3

The relation between P fertilization rate and YI is evaluated here in terms of relative rates, i.e., P input divided by the P export by the harvested crop (Fig. 4). Although the YI values are on average highest for relative rates of 100–150%, the differences among the groups are mostly not significant, and conspicuously, the YI values are relatively low for high rates of P input (> 200% of exported P). This applies also when only data for soil P class B are considered (not shown here in detail).

Such a lack of stringent relation between P fertilizer amount and yield increase has been reported in previous studies (e.g., Jungk et al. 1993) and suggests that in most cases the pool of plant-available P in the soil is sufficient for high crop yields, and the applied fertilizer P is used mainly to maintain or even enhance this soil P pool. This is in accordance with the philosophy of “maintenance fertilization” (Jordan-Meille et al. 2012), although recently this approach has been questioned (Withers et al. 2014). Additionally, more important than the applied amount of fertilizer is the P content of the control plot. In cases where the soil P content of the unfertilized control is above 9 mg P/100 g, the fertilized plots only show an average yield increase of 1.1%, irrespective of the total available P (soil P + fertilizer P) in the treatment plot. A low correlation between P amount supplied versus plant yields (Pearson *r* = −0.10***) may also indicate the active mobilization of soil P resources by plants by root exudates, mycorrhiza and fine roots (Eichler-Löbermann et al. 2007; Requejo and Eichler-Löbermann 2014), which is not routinely measured and is not a part of current fertilization recommendations.

When evaluating the effect of P fertilizer type on YI, there are statistically significant differences between treatments fertilized with Superphosphate and Thomas phosphate on one hand, and Triple superphosphate and Hyperphosphate on the other hand (Table 3). The lower effectiveness of Hyperphosphate (i.e., finely ground rock phosphate) in non-acid soils compared with Superphosphate is expected and in line with previous studies (e.g., Spiegel et al. 2001; von Tucher 2013). However, one would expect that Superphosphate and Triple superphosphate are similarly available, since both are produced by reaction of rock phosphate with inorganic acids (sulfuric acid and phosphoric acid). In contrast to Triple superphosphate, Superphosphate contains remnants of sulfate, which is a macronutrient. This could be an explanation for the higher effectiveness of Superphosphate. The relatively high effectiveness of Thomas phosphate could possibly be caused by the high content of Ca and micronutrients (e.g., Fe, Mn, Zn), and the alkaline soil reaction induced by this fertilizer.

**Fig. 5:**
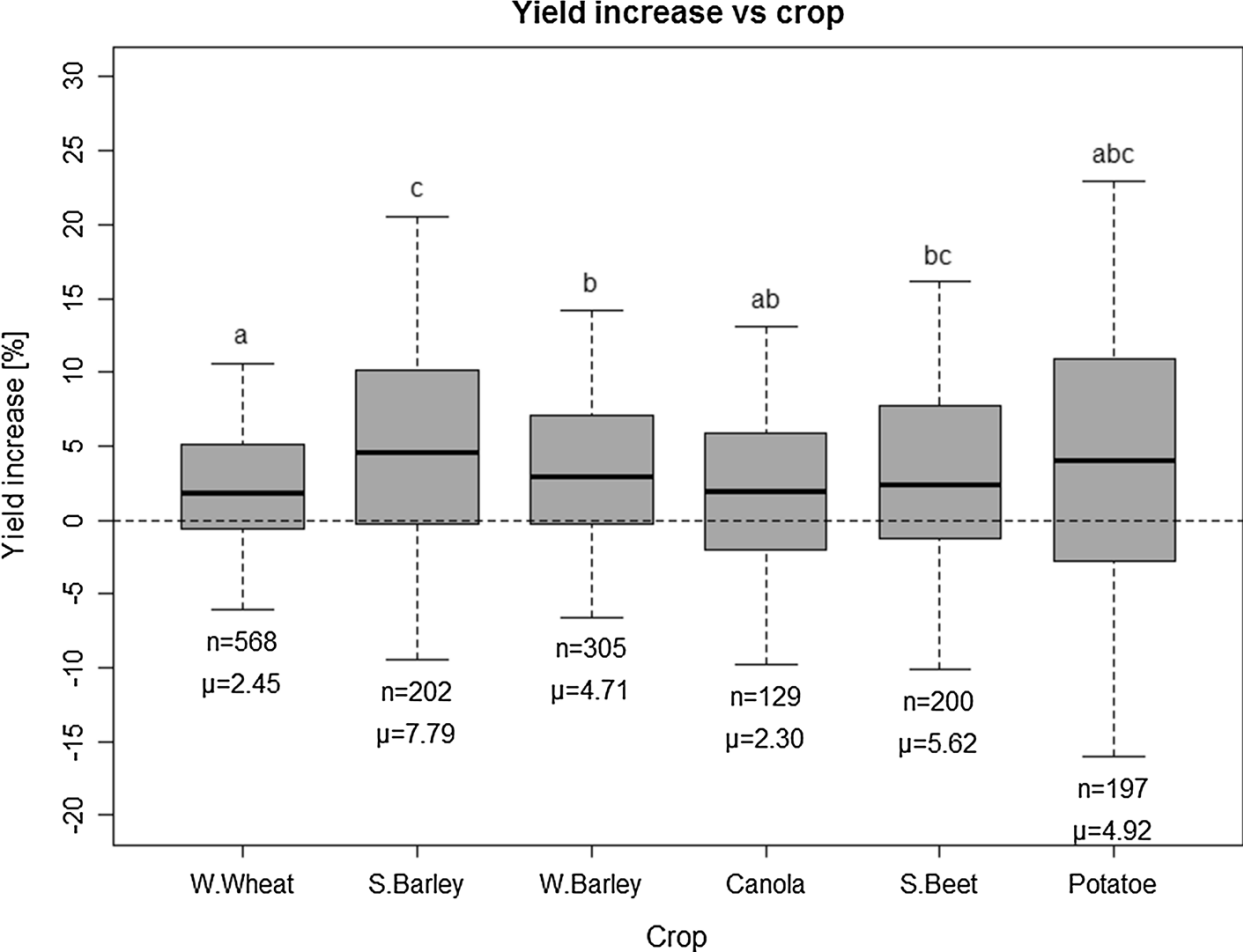
Rel. yield increase (YI) versus crop type; for the meaning of symbols, please refer to Fig. 3

**Fig. 6:**
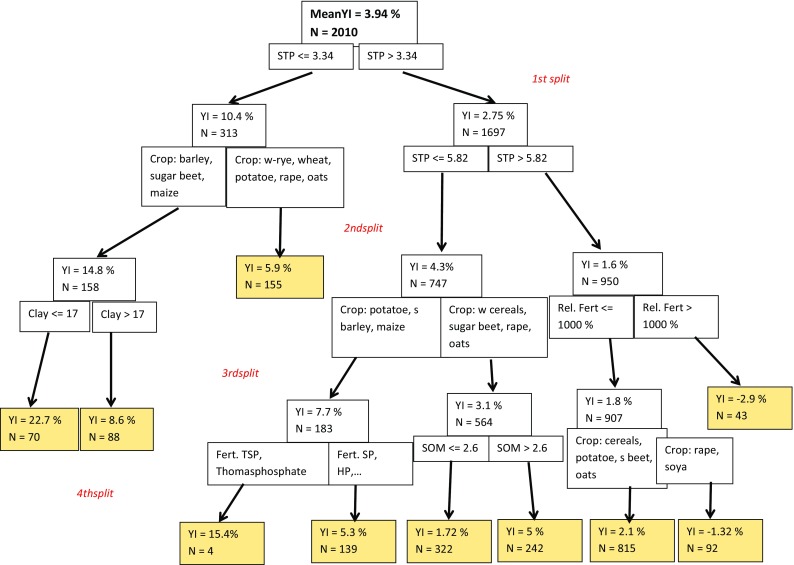
CART analysis of rel. yield increase. Considered independent variables are STP, pH, SOM, clay content, rel. P fertilizer amount (%), crop type, and fertilizer type; not all these independent variables appear in the graph

A comparison of the effectiveness of P fertilization among the six most common crops (Fig. 5) shows overall highest yield increase for summer barley, and lowest increases for winter wheat. When only soils with low soil phosphorus content (fertility class B) are considered, sugar beet shows the strongest response (12.6% mean YI) to fertilizer application, winter wheat (3.2% mean YI) and canola (oilseed rape) (2.7% mean YI) tend to respond less (not shown here in detail). In the previous section (Table 3 and Figs. 2, 3, 4, 5), the YI was evaluated as a function of several separate factors. All these factors are combined as independent variables in an analysis by means of a classification and regression tree approach (Fig. 6).

The first split was set by the CART algorithm for the independent variable plant-available soil P content (STP), at a value of 3.34 mg P 100 g^−1^ soil. This indicates that plant-available soil P content is the most important variable determining yield increase by P fertilization.

If the STP of the control is above 3.3 mg P 100 g^−1^, average YI is only 2.75% (compared to 10.4%). This result supports the latest VDLUFA recommendation (VDLUFA 2015) to reduce the lower boundary of the P content class “C” to 3.0 mg P 100 g^−1^.

The second split is implemented according to crop species and again STP, i.e., these independent variables explain for each of the branches the largest part of the variance in YI. The blue end segments indicate the mean YI for the combination of parameters according to the respective branch of the decision tree. This can be demonstrated exemplarily for a dataset from Rottenhaus (Austria) dating from the year 1981 (Spiegel et al. 2001). The plant-available soil P content is 4.5 mg P 100 g^−1^ soil, i.e., at the lower margin of P content class C (VDLUFA 1997). Clay content is 30% and pH 5.98. The fertilizer application rate of 172 kg P ha^−1^ year^−1^ in the form of Superphosphate corresponds to 642% of P export by the crop (26.8 kg P ha^−1^). Nevertheless, the fertilizer application rate is, according to the CART analysis, not among the most important variables explaining the observed yield increase. For this dataset, the predicted YI is 5.3% (Fig. 6), whereas measured YI is 4.5%. Similarly, multiple linear regression analysis suggests that plant-available P content, pH value, and SOM content are the most significant variables; however, with large differences among different fertilizer types (Table 4). This is in line with the results from a meta-analysis of P fertilizer experiments in Finland (Valkama et al. 2009).

**Table 4:** Results of multiple linear regression analysis of rel. yield increase as a function of pH, P(CAL,DL), SOM content, clay content, and relative fertilizer amount; all data combined and separately for fertilizer types

Parameter	Standardized coefficient (*β*)			
	All data	Superphosphate	TSP	Thomas phosphate
pH	− 0.111***	0.044 (ns)	− 0.162***	0.112 (ns)
P(CAL,DL)	− 0.152***	− 0.374***	− 0.078 (ns)	− 0.206**
SOM	0.097***	0.023 (ns)	0.17**	0.213**
Clay content	− 0.033 (ns)	− 0.183***	− 0.072 (ns)	− 0.228**
Relative fertilizer amount	− 0.021 (ns)	0.16**	0.055 (ns)	− 0.171*

Significance levels: * *p* < 0.05; ** *p* < 0.01; *** *p* < 0.001; ns: *p* > 0.05 (not significant)

## Conclusions

This meta-analysis of a database of long-term field experiments of P fertilization covering various regions of Germany and Austria including about 2000 datasets from 30 field sites revealed that yield increase due to the effect of fresh P application is determined mainly by plant-available P in the soil, pH value, SOM, type of fertilizer, and crop type, whereas the exact amount of P fertilizer has less importance. The database will be expanded in the near future, and additional parameters will be included in the analysis, most notably soil type, precipitation, and air temperature. In a next step, the results will be utilized to refine the current P fertilizer recommendations. Although only data from Germany and Austria are utilized in the present analysis, this approach can be extended to other countries worldwide, and the results gained in the analyses can be transferred to other environmental conditions and countries. This could contribute to more precise P fertilization recommendations, less application of P fertilizer, and diminished negative environmental impacts of P fertilization.

## Electronic supplementary material

Below is the link to the electronic supplementary material.

Supplementary material 1 (PDF 508 kb)
