# Supplementary material for: Re-evaluation of the yield response to phosphorus fertilization based on meta-analyses of long-term field experiments
Source: Ambio. 2017 Nov 20;47(Suppl 1):50–61. doi: 10.1007/s13280-017-0971-1 (PMC5722740; doi:10.1007/s13280-017-0971-1)
Supplement: Supplementary file 1 — Supplementary material 1 (PDF 508 kb) [file 13280_2017_971_MOESM1_ESM.pdf]

*Ambio*

**Electronic Supplementary Material**

**Title: Re-evaluation of the yield response to phosphorus fertilization based on meta-analyses of long-term field experiments**

Authors: Uwe Buczko, Michael van Laak, Bettina Eichler-Löbermann, Wolfgang Gans, Ines Merbach, Kerstin Panten, Edgar Peiter, Thomas Reitz, Heide Spiegel, Sabine v. Tucher

**Table S1** Details about the institutions performing the field experiments and the corresponding field sites

| **Institute** | **Contact** | **Field experiment** | **description** | **References** |
|---|---|---|---|---|
| Helmholtz-Centre for Environmental Research (Helmholtz-Zentrum für Umweltforschung – UFZ) | Dr. Ines Merbach (ines.merbach@ufz.de) | Static Fertilization Experiment Bad Lauchstädt | Experiment conducted since 1902, focus on the effects of mineral and organic fertilizers on ecosystem processes and services, 18 different fertilization treatments | Merbach and Schulz (2012) |
| Mecklenburg-Vorpommern Research Centre for Agriculture and Fisheries (Landesforschungsa | Dr. Ines Bull (i.bull@lfa.mvnet.de) | Static P/K-Fertilization Experiment Gülzow | start in 1997, rotation: winter rape – winter wheat – | Boelcke (2007) |

| | | | | |
|---|---|---|---|---|
| nstalt für Landwirtschaft und Fischerei Mecklenburg-Vorpommern) | | | potato – winter barley, treatments with different P- and K-fertilization, only mineral fertilizers | |
| Agricultural research station Kamperhof (Landwirtschaftliche Versuchsanstalt Kamperhof) Mülheim / Ruhr | Dr. Martin Rex (rex@hk-kalke.de) | Hohensolms, Lehsen, Sieversen | Since 1994; fertilizer type, application in autumn or spring | Rex (1999) |
| Chair of Plant Nutrition, TU Munich (Lehrstuhl für Pflanzenernährung TU München) | Dr. Sabine von Tucher (tucher@wzw.tum.de) | Static Research Station Freising-Dürnast 016+021/022 | 016 since 1978 application of P fertilizer and lime; 021/022 since 1979 application | von Tucher et al. (2016) |

| | | | | |
|---|---|---|---|---|
| | | | of P, mineral N and organic matter | |
| Austrian Agency for Health and Food Safety (Österreichische Agentur für Gesundheit und Ernährungssicherheit) | Dr. Heide Spiegel (adelheid.spiegel@ages.at) | Three long-term P-field experiments in Lower Austria (Fuchsenbigl, Rottenhaus and Zwettl) | Long-term P-field experiments conducted from 1956 to 2004 (Zwettl) / to 2014 (in Fuchsenbigl and Rottenhaus) studying the effects of different types (Super-P, Thomas-P, Hyper-P and quantities of P- | Spiegel et al. (2001); Lindenthal et al. (2003) |

| | | | | |
|---|---|---|---|---|
| | | | fertilisation on crop and soil parameters | |
| Plant Nutrition Laboratory, Institute of Agricultural and Nutritional Sciences, Martin Luther University Halle - Wittenberg | Prof. Dr. Edgar Peiter (edgar.peiter@landw.uni-halle.de), Dr. Wolfgang Gans (wolfgang.gans@landw.uni-halle.de) | Phosphate Fertilization Experiment (Field D), J. Kühn-Field, Halle (Saale) | Experiment initiated 1949 by Karl Schmalfuß; Focus on different annual and triennial P application rates combined with the comparison of superphosphate and basic slag | Gransee and Merbach (2000) |
| Julius Kühn Institute (JKI), Dept for Crop and Soil Science; | Dr. Kerstin Panten (kerstin.panten | Long-term P-fertilization experiment in | From 1985-1997; Variables: | Vogeler et al. (2009) |

| | | | | |
|---|---|---|---|---|
| Julius Kühn-Institut (JKI), Institut für Pflanzenbau und Bodenkunde | @julius-kuehn.de) | Braunschweig | fertilizer type, amount, time of application | |
| Agricultural technology center Augustenberg (Landwirtschaftliches Technologiezentrum Augustenberg), Karlsruhe | Dr. Markus Mokry markus.mokry@ltz.bwl.de) | Blaufelden, Schw. Gmünd, Emmendingen, Tuttlingen, Pfullendorf, Biberach, Ladenburg | Duration 1984 – 1993 Objective effectiveness of P-fertilization as a function of soil P and class divisions | Mokry (1996) |
| Landesbetrieb Landwirtschaft Hessen | Dr. Johannes Heyn / Dierk Koch dierk.koch@llh.hessen.de | 11 sites in Hesse (see Table 1) | Various duration, starting in 1974; effectiveness of P fertilization as function of soil P | Schaumberg and Heyn (1996) |

| | | | content | |
|---|---|---|---|---|
| University of Rostock, Chair Agronomy | Prof. Bettina Eichler-Löbermann (bettina.eichler@uni-rostock.de) | since 1998 - split plot design whereas organic fertilization constitutes the main plots (applied every third year), mineral P fertilization constitutes the sub plots (applied every year), and management practice constitutes the sub-sub plots - three levels of organic fertilizers (zero, cattle manure, | Long-term P management strategies including organic and inorganic fertilizers | Requejo & Eichler-Löbermann (2014), Krey et al. (2013), Eichler-Löbermann et al. (2007) Eichler-Löbermann et al. (2008) |

| | | | | |
|---|---|---|---|---|
| | | biowaste compost), three levels of inorganic fertilizers (zero, TripleSuper autumn application, TripleSuper spring application/biomass ash) | | |
